# Association Between Age and Severity of Acute Appendicitis in Adults

**DOI:** 10.3390/life16081266

**Published:** 2026-07-31

**Authors:** Liviu Vasile, Laurențiu Augustus Barbu, Nicolae-Dragoș Mărgăritescu, Stelian-Stefaniță Mogoantă, Tiberiu Stefăniță Țenea Cojan, Gabriel Florin Răzvan Mogoș

**Affiliations:** 1 Department of Surgery, Emergency County Hospital, University of Medicine and Pharmacy of Craiova, 2 Petru Rares Street, 200349 Craiova, Romania; vliviu777@yahoo.com (L.V.); dmargaritescu@yahoo.com (N.-D.M.); ssmogo@yahoo.com (S.-S.M.); 2 Department of Surgery, Railway Clinical Hospital Craiova, University of Medicine and Pharmacy of Craiova, 2 Petru Rares Street, 200349 Craiova, Romania; tiberiu.tenea@umfcv.ro (T.S.Ț.C.); gabriel.mogos@umfcv.ro (G.F.R.M.)

**Keywords:** acute appendicitis, complicated appendicitis, age, elderly patients, disease severity, inflammatory markers, retrospective study, appendectomy, emergency surgery

## Abstract

**Background**: Early identification of complicated appendicitis remains challenging, particularly in elderly patients who often present with atypical symptoms and delayed diagnosis. This study evaluated the association between age, inflammatory markers, and the severity of acute appendicitis. **Methods**: This retrospective comparative study included 311 adult patients (182 males, 58.5%, and 129 females, 41.5%) who underwent appendectomy between January 2020 and December 2024. Demographic, clinical, and laboratory data were analyzed. Logistic regression and receiver operating characteristic (ROC) analyses were performed to assess the association between age and complicated appendicitis. **Results**: Complicated appendicitis was identified in 120 patients (38.6%). Patients with complicated appendicitis were significantly older than those with uncomplicated disease (49.53 ± 16.85 vs. 39.53 ± 17.58 years, *p* < 0.001). In the prespecified multivariable logistic regression model including age, sex, leukocyte count, and C-reactive protein (CRP), increasing age remained significantly associated with complicated appendicitis (adjusted OR 1.25 per 10-year increase, 95% CI 1.10–1.45, *p* = 0.002). Leukocyte count (adjusted OR 1.05 per 1000/mm^3^ increase, 95% CI 1.02–1.08, *p* < 0.001) and CRP (adjusted OR 1.12 per 10 mg/L increase, 95% CI 1.08–1.16, *p* < 0.001) were also significantly associated with complicated appendicitis in the multivariable model, whereas male sex was not. ROC analysis showed poor-to-modest discriminatory ability of age for distinguishing uncomplicated from complicated appendicitis (AUC = 0.669, 95% CI: 0.609–0.731), indicating that age alone has limited value as a standalone predictor. **Conclusions**: Although increasing age remained significantly associated with complicated appendicitis after adjustment for sex, leukocyte count, and C-reactive protein (CRP), the adjustment was limited to variables consistently available in this retrospective dataset. Therefore, residual confounding cannot be excluded, and the observed association should not be interpreted as evidence of a fully independent effect. Furthermore, the discriminatory performance of age was only modest (AUC = 0.669), indicating that age should not be used as a standalone predictor and should be interpreted within the overall clinical context.

## 1. Introduction

Acute appendicitis remains one of the most common surgical emergencies worldwide, with a lifetime risk estimated between 6% and 9% [1]. Despite its high prevalence, the clinical presentation is highly variable, ranging from mild, self-limiting inflammation to severe forms such as perforation, abscess formation, and generalized peritonitis [2,3]. Distinguishing between uncomplicated and complicated appendicitis is clinically important because disease severity is associated with increased morbidity, mortality, and healthcare resource utilization.

Although advances in diagnostic evaluation have improved the management of acute appendicitis, delayed or missed diagnosis remains a significant clinical challenge. Diagnostic errors and delayed presentation have been associated with disease progression, perforation, sepsis, prolonged hospitalization, and postoperative complications [4,5,6,7,8]. Early identification of patients at increased risk of complicated appendicitis may therefore facilitate timely intervention and improve clinical outcomes.

Age has been consistently recognized as an important factor associated with disease severity. Older patients are more likely to present with advanced forms of appendicitis, possibly because of atypical clinical manifestations and delayed diagnosis [9,10]. In addition, inflammatory markers such as leukocyte count and C-reactive protein (CRP) are routinely used in the evaluation of patients with suspected appendicitis. However, their relationship with disease severity remains inconsistent across studies, and their ability to identify complicated appendicitis remains uncertain [11,12].

Although the association between older age and complicated appendicitis has been consistently reported, it remains uncertain whether age provides clinically meaningful discriminatory value beyond routinely available inflammatory markers. Most previous studies have primarily focused on demonstrating statistical associations between age and disease severity, whereas the practical predictive performance of age for identifying complicated appendicitis has received less attention. Furthermore, published findings regarding the association of leukocyte count and C-reactive protein (CRP) with disease severity remain inconsistent, and evidence from contemporary adult surgical cohorts is still limited. Therefore, further evaluation of these readily available parameters may provide additional information for clinical risk stratification in patients with acute appendicitis.

Given these considerations, the present study aimed to provide additional confirmatory evidence from a contemporary single-center adult surgical cohort regarding the association between age and complicated appendicitis. We also evaluated the discriminatory ability of age and explored the associations of routinely available inflammatory markers, including leukocyte count and C-reactive protein (CRP), with disease severity.

## 2. Materials and Methods

### 2.1. Study Design and Population

**Figure 1:**
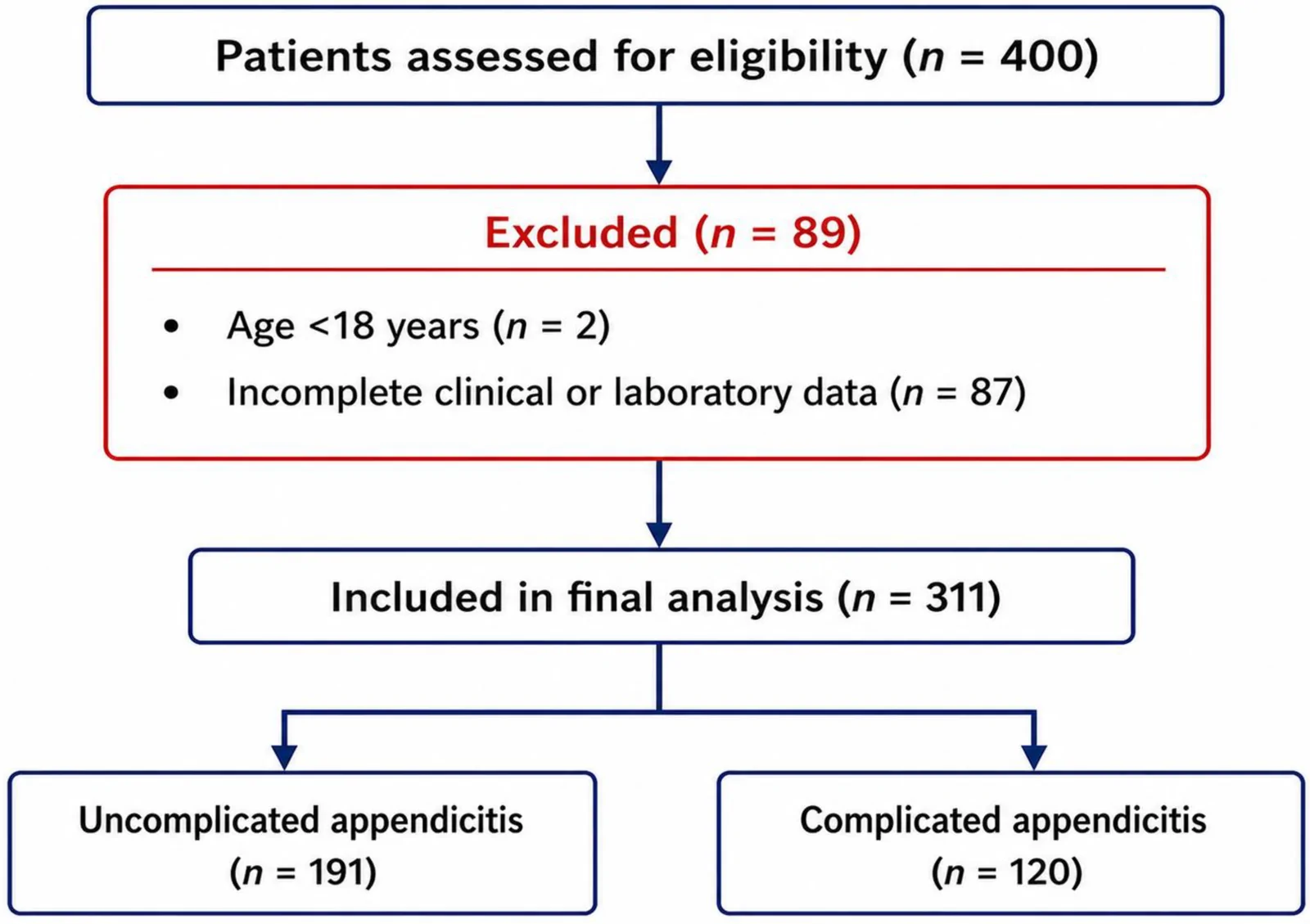
Flowchart of patient selection, exclusions, and group allocation.

This retrospective single-center cohort study included adult patients diagnosed with acute appendicitis who underwent appendectomy at the Emergency County Clinical Hospital Craiova, Romania. Patients were treated in one of the three Departments of Surgery (I, II, and III) within the same hospital between January 2020 and December 2024 (Figure 1).

Appendectomy was performed according to standard institutional practice, using either laparoscopic or open approach, depending on patient characteristics, disease severity, and surgeon preference.

Inclusion criteria comprised patients aged ≥18 years with a confirmed diagnosis of acute appendicitis who underwent surgical treatment. Patients with incomplete clinical or laboratory data were excluded from the study. Additional exclusion criteria such as comorbidities, prior antibiotic use, inflammatory or malignant diseases, and pregnancy were not systematically applied due to the retrospective nature of the study. These factors were not consistently available in the medical records and may have influenced the investigated parameters.

### 2.2. Data Collection

Clinical and demographic data were retrospectively extracted from electronic medical records. The following variables were collected: age, sex, type of appendicitis (complicated vs. uncomplicated), leukocyte count, and C-reactive protein (CRP) levels at admission.

Histopathological examination was routinely performed in all patients to confirm the diagnosis of acute appendicitis. Classification into complicated and uncomplicated appendicitis was based on predefined intraoperative findings documented in the surgical reports. Complicated appendicitis was defined as the presence of appendiceal perforation, abscess formation, or generalized peritonitis, whereas uncomplicated appendicitis included cases without these features. Gangrenous appendicitis, localized peritonitis, appendiceal phlegmon, and contained perforation were not recorded as separate diagnostic categories in the surgical reports available for retrospective review. Consequently, patients were classified according to the predefined study definition based on the documented presence of perforation, abscess formation, or generalized peritonitis. These predefined criteria were established before data extraction and applied uniformly across all three surgical departments of the Emergency County Clinical Hospital Craiova. Because of the retrospective nature of the study, blinding of assessors to patient age or laboratory values was not feasible.

### 2.3. Study Objectives and Outcome

The primary objective of the study was to evaluate the association between age and the presence of complicated appendicitis in adult patients.

The primary outcome was the presence of complicated appendicitis, defined according to the predefined intraoperative criteria described above.

Secondary objectives were to assess the associations of routinely available inflammatory markers (leukocyte count and C-reactive protein [CRP]) with complicated appendicitis and to evaluate the discriminatory ability of age for identifying complicated disease using receiver operating characteristic (ROC) curve analysis.

### 2.4. Statistical Analysis

The normality of continuous variables was assessed using the Shapiro–Wilk test. Normally distributed variables were expressed as mean ± standard deviation (SD) and compared using Student’s *t*-test. Leukocyte count and C-reactive protein (CRP) showed non-normal distributions and were therefore summarized as median (interquartile range [IQR]) and compared using the Mann–Whitney U test. Categorical variables were presented as frequencies and percentages and compared using the chi-square test. A prespecified multivariable logistic regression model was constructed with complicated appendicitis as the dependent variable. Age, sex, leukocyte count, and C-reactive protein (CRP) were entered simultaneously into the model based on their clinical relevance, irrespective of their statistical significance in univariable analyses. Age was modeled as a continuous variable per 10-year increase, leukocyte count per 1000/mm^3^ increase, and CRP per 10 mg/L increase. Female sex was used as the reference category. Adjusted odds ratios (ORs) with 95% confidence intervals (CIs) were reported. Although leukocyte count and CRP were not normally distributed, they were analyzed on their original continuous scale because logistic regression does not require predictor variables to be normally distributed. The original scale was retained to facilitate clinical interpretation of the estimated odds ratios; although log-transformation represents an alternative modelling strategy for skewed continuous variables, it was not applied in the present study. The assumption of linearity in the logit for the continuous predictors (age, leukocyte count, and CRP) was evaluated using the Box–Tidwell procedure. Interaction terms between each continuous predictor and its natural logarithmic transformation were included in the model. A Bonferroni-adjusted significance level (*p* < 0.0167) was used to account for multiple testing. Multicollinearity among the predictors was assessed using variance inflation factors (VIFs).

Receiver operating characteristic (ROC) curve analysis was performed to evaluate the ability of age to differentiate between uncomplicated and complicated appendicitis. The area under the curve (AUC) was calculated to evaluate the discriminatory ability of age for differentiating between uncomplicated and complicated appendicitis. A *p*-value < 0.05 was considered statistically significant. Statistical analyses were performed using IBM SPSS Statistics version 26.0 (IBM Corp., Armonk, NY, USA).

Due to the retrospective design of the study, no a priori sample size calculation was performed.

### 2.5. Ethical Considerations

The study was conducted in accordance with the Declaration of Helsinki and was approved by the Ethics Committee of the Emergency County Clinical Hospital Craiova (approval no. 14827, 20 March 2026). Due to the retrospective design of the study, the requirement for informed consent was waived.

## 3. Results

A total of 311 patients were included in the study. The mean age was 43.28 ± 17.97 years, ranging from 18 to 86 years. The cohort included 182 males (58.5%) and 129 females (41.5%). Complicated appendicitis was identified in 120 patients (38.6%), while 191 patients (61.4%) had uncomplicated disease. Baseline characteristics and comparisons between patients with uncomplicated and complicated appendicitis are presented in Table 1. Complicated appendicitis was further classified according to disease severity. Patients with complicated appendicitis were significantly older than those with uncomplicated disease (49.53 ± 16.85 vs. 39.53 ± 17.58 years, *p* < 0.001). No significant differences in sex distribution were observed between the two groups (*p* = 0.591).

Multivariable logistic regression analysis including age, sex, leukocyte count, and C-reactive protein (CRP) showed that increasing age was significantly associated with complicated appendicitis in the multivariable model. Each 10-year increase in age was associated with a 25% increase in the odds of complicated appendicitis (adjusted OR 1.25, 95% CI 1.10–1.45, *p* = 0.002). Leukocyte count (adjusted OR 1.05 per 1000/mm^3^ increase, 95% CI 1.02–1.08, *p* < 0.001) and CRP (adjusted OR 1.12 per 10 mg/L increase, 95% CI 1.08–1.16, *p* < 0.001) were also significantly associated with complicated appendicitis in the multivariable model, whereas male sex was not significantly associated with disease severity (adjusted OR 1.10, 95% CI 0.70–1.70, *p* = 0.65) (Table 2).

Although leukocyte count and CRP were not significantly different between groups in the univariable comparisons (Table 3), both variables remained significantly associated with complicated appendicitis after adjustment for age and sex in the multivariable model. This difference likely reflects the independent contribution of these variables after simultaneous adjustment for the other covariates included in the regression model. Unlike the univariable analysis, which evaluates each predictor separately, the multivariable model estimates the independent association of each variable while controlling for the remaining predictors. Therefore, variables that are not statistically significant in univariable analyses may become significant after adjustment because the influence of confounding among the included variables is reduced.

The assumption of linearity in the logit was assessed using the Box–Tidwell procedure. No significant departures from linearity were observed for age, leukocyte count, or CRP (all Bonferroni-adjusted *p* > 0.0167).

Assessment of multicollinearity showed no evidence of relevant collinearity among the predictors (all VIF values < 2).

**Figure 2:**
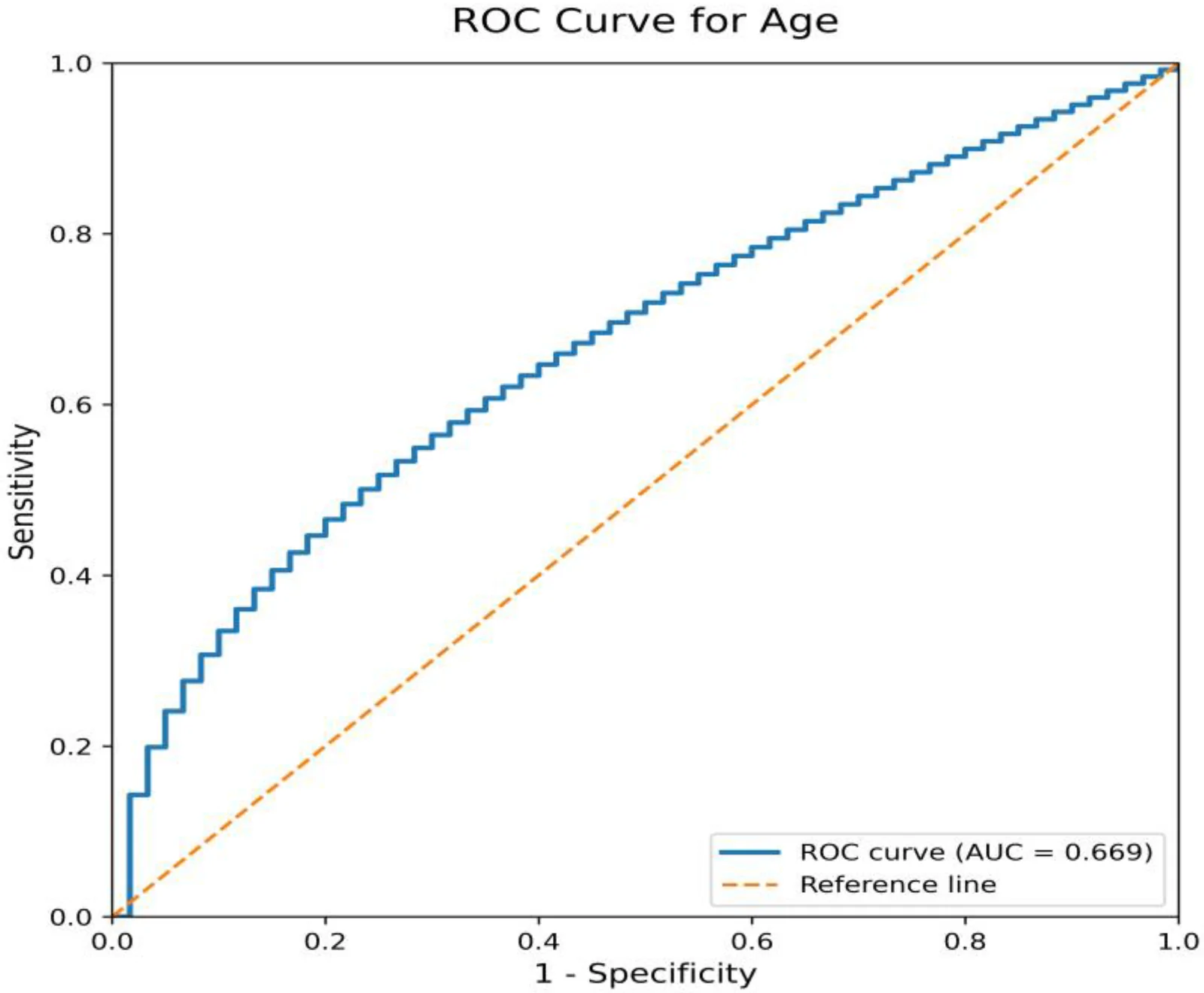
Receiver operating characteristic (ROC) curve showing the discriminatory ability of age for distinguishing complicated from uncomplicated appendicitis. The area under the curve was 0.669 (95% CI: 0.609–0.731), indicating poor-to-modest discrimination.

ROC analysis showed poor-to-modest discriminatory ability of age for distinguishing complicated from uncomplicated appendicitis (AUC = 0.669, 95% CI: 0.609–0.731; Figure 2). These findings indicate that age alone has limited discriminatory ability and should not be used as a standalone predictor. Because the multivariable model was restricted to variables consistently available in the retrospective dataset (age, sex, leukocyte count, and C-reactive protein [CRP]), the adjusted associations should be interpreted with caution. Important potential confounders, including symptom duration, comorbidities, imaging findings, prior antibiotic treatment, and time to surgery, were unavailable and therefore could not be included in the model. Consequently, residual confounding cannot be excluded.

To further explore whether the association between age and complicated appendicitis differed across clinically relevant age categories, an exploratory descriptive analysis was performed (Table 4). The frequency of complicated appendicitis was higher among patients aged 40 years or older than among those younger than 40 years (31.0% vs. 45.7% and 46.9%, respectively). The similar frequencies observed in the 40–59-year and ≥60-year groups suggest a possible threshold effect rather than a progressive increase across older age categories. Because no formal trend analysis was performed, this observation is exploratory and should be interpreted cautiously.

Baseline laboratory parameters are presented in Table 3. Leukocyte count and C-reactive protein (CRP) were not normally distributed according to the Shapiro–Wilk test and are therefore presented as median (interquartile range, IQR). No significant differences were observed between patients with uncomplicated and complicated appendicitis for leukocyte count {14,200 (2,100–16,800) vs. 14,800 (12,700–16,500) cells/mm^3^, Mann–Whitney U test, *p* = 0.29} or CRP {27 (16–48) vs. 31 (19–54) mg/L, Mann–Whitney U test, *p* = 0.39).

## 4. Discussion

### 4.1. Principal Findings

The principal finding of the present study is not merely that increasing age is associated with complicated appendicitis, an observation consistently reported in previous studies, but that age alone demonstrated only modest discriminatory ability (AUC = 0.669). This finding suggests that, although age remained significantly associated with complicated appendicitis after adjustment for sex, leukocyte count, and C-reactive protein (CRP), it should not be interpreted as a standalone clinical predictor. Instead, age should be considered together with laboratory parameters and the overall clinical assessment when estimating the likelihood of complicated disease.

In our multivariable model, increasing age remained significantly associated with complicated appendicitis after adjustment for sex, leukocyte count, and CRP. Leukocyte count and CRP also remained significantly associated with complicated appendicitis after adjustment for the other variables included in the multivariable model, whereas male sex was not. However, the interpretation of these associations should be made with caution. Because several clinically important variables—including symptom duration, comorbidities, imaging findings, prior antibiotic treatment, and time to surgery—were unavailable, the multivariable model could adjust only for sex, leukocyte count, and CRP. Consequently, residual confounding cannot be excluded, and the observed associations should not be interpreted as evidence of fully independent causal effects.

These findings are consistent with previous reports showing that older patients are more likely to present with perforation, abscess formation, or generalized peritonitis. Several mechanisms may contribute to this observation, including atypical clinical presentation, delayed diagnosis, reduced physiological reserve, and the higher prevalence of comorbidities among elderly individuals.

### 4.2. Comparison with Previous Studies and Interpretation

The findings of the present study are consistent with the existing literature, confirming that acute appendicitis represents a heterogeneous condition with variable clinical severity, ranging from uncomplicated inflammation to perforation and generalized peritonitis [13,14,15,16,17,18,19]. This variability in presentation has important clinical implications, as complicated appendicitis is associated with significantly higher morbidity, mortality, and healthcare resource utilization.

A key finding of this study is the significant association between age and disease severity. This finding is in agreement with previous studies demonstrating that older patients are more likely to present with advanced stages of appendicitis and have an increased risk of complications, including perforation and peritonitis [20,21,22,23].

Several mechanisms have been proposed in previous studies to explain the association between older age and complicated appendicitis, including atypical clinical presentation, altered inflammatory response, delayed healthcare-seeking behavior, and delayed diagnosis. These factors have been associated with an increased risk of perforation, prolonged hospitalization, and worse postoperative outcomes in older adults [24,25,26,27]. However, because symptom duration, healthcare access, and diagnostic delay were not evaluated in the present study, these mechanisms should be regarded as plausible explanations reported in the literature rather than findings directly supported by our data.

Patients aged 40 years or older showed a higher frequency of complicated appendicitis than those younger than 40 years. However, the frequencies were similar in the 40–59-year and ≥60-year groups (45.7% vs. 46.9%), suggesting a possible threshold effect rather than a progressive increase across age categories. Because no formal trend analysis was performed, this observation should be considered exploratory and requires confirmation in larger prospective studies. Previous studies have suggested that delayed presentation and healthcare-system factors may contribute to the development of complicated appendicitis; however, these variables were not evaluated in the present study [16,23,28,29,30,31,32,33,34].

Another important finding of the present study is the lack of association between sex and disease severity, which is consistent with prior studies suggesting that demographic variables other than age may have a relatively minor role in disease progression.

An interesting finding of the present study was that, although leukocyte count and CRP did not differ significantly between groups in the univariable analysis, both variables remained significantly associated with complicated appendicitis after adjustment for the other variables included in the multivariable model. Similar associations between elevated inflammatory markers and complicated appendicitis have been reported in several previous studies, whereas others have found inconsistent or limited predictive value. These discrepancies may reflect differences in patient selection, definitions of complicated appendicitis, timing of laboratory testing relative to symptom onset, and adjustment for potential confounding factors. Inflammatory marker levels are dynamic and may vary substantially according to the duration of the inflammatory process; therefore, measurements obtained at hospital admission may not fully capture disease severity in all patients. Furthermore, multivariable regression evaluates the independent contribution of each variable after adjustment for other predictors, which may explain why leukocyte count and CRP were significant in the adjusted analysis despite the absence of significant differences in the univariable comparison. Taken together, our findings support the use of inflammatory markers as complementary components of clinical risk assessment rather than as standalone predictors of complicated appendicitis.

### 4.3. Clinical Implications and Novelty

Rather than demonstrating a novel association between age and complicated appendicitis, our study reinforces existing evidence while emphasizing the distinction between statistical association and clinical usefulness. The findings support interpreting age as one component of a comprehensive clinical assessment, alongside inflammatory markers and other clinical factors, rather than as an isolated predictor.

The multivariable analysis further showed that leukocyte count and CRP remained significantly associated with complicated appendicitis after adjustment for age and sex. These findings suggest that routinely available laboratory markers may provide complementary information when interpreted together with age, although their clinical utility remains to be established. Nevertheless, none of these variables should be considered in isolation, and clinical judgment remains essential.

Finally, while surgical timing has traditionally been considered a key determinant of outcome, recent evidence suggests that disease severity is more closely related to the duration of symptoms prior to presentation rather than in-hospital delay. Although the present study did not evaluate symptom duration or diagnostic delay, previous evidence indicates that prolonged prehospital symptom duration is associated with disease progression.

Taken together, these findings provide additional confirmatory evidence supporting the previously reported association between increasing age and complicated appendicitis. However, the discriminatory performance of age alone was only modest (AUC = 0.669), indicating that these findings should be interpreted cautiously and require further prospective validation before clinical application.

### 4.4. Strengths and Limitations

This study has several strengths. It included a real-world cohort of adult patients treated in routine clinical practice over a five-year period. Age was evaluated both as a continuous variable and across clinically relevant age groups, providing complementary descriptive information regarding disease severity. These strengths should be interpreted in the context of the study’s retrospective single-center design and the limitations described below.

However, several limitations should be acknowledged. Due to the retrospective design of the study, data on body mass index (BMI) and American Society of Anesthesiologists (ASA) score were not consistently available and were therefore not included in the analysis. The retrospective single-center design introduces the potential for selection bias and limits the generalizability of the findings. In addition, 87 patients were excluded because of incomplete clinical or laboratory data. Because the excluded patients had incomplete records, a formal comparison between included and excluded patients could not be reliably performed. Therefore, selection bias cannot be excluded. Furthermore, only surgically treated patients were included, which may have influenced the study population.

The multivariable model included age, sex, leukocyte count, and C-reactive protein (CRP). However, several important potential confounders, including comorbidities, prior antibiotic use, symptom duration, time to surgery, pregnancy status, inflammatory or malignant diseases, ASA status, and imaging findings, were not consistently available and therefore could not be included in the analyses. Because these factors may be associated with both patient age and appendicitis severity, residual confounding cannot be excluded. Consequently, the observed associations should not be interpreted as evidence of fully independent effects but rather as exploratory findings requiring confirmation in larger prospective multicenter studies.

Although receiver operating characteristic analysis was performed to assess model discrimination, model calibration was not formally evaluated because the objective of the present study was to investigate associations rather than to develop or validate a clinical prediction model. Therefore, the predictive performance of the model should be interpreted with caution.

Postoperative outcomes, including intra-abdominal abscess formation, were not systematically evaluated in this study. In addition, the impact of surgical approach (laparoscopic versus open appendectomy) on postoperative complications was not analyzed. These aspects represent important areas for future research.

## 5. Conclusions

In this retrospective single-center cohort, increasing age remained significantly associated with complicated appendicitis after adjustment for sex, leukocyte count, and C-reactive protein (CRP). However, because adjustment was limited to variables consistently available in this retrospective dataset, residual confounding cannot be excluded, and these findings should be interpreted as exploratory rather than as evidence of a fully independent association. Furthermore, ROC analysis demonstrated only modest discriminatory performance (AUC = 0.669), indicating that age alone is insufficient for clinical prediction and should be interpreted together with routinely available laboratory markers and the overall clinical assessment. Leukocyte count and CRP were also significantly associated with disease severity after adjustment, although these findings require prospective validation before clinical application. Larger prospective multicenter studies incorporating additional clinically relevant variables are needed to validate these findings and to develop more comprehensive prediction models for complicated appendicitis.

## Figures and Tables

**Table 1:** Baseline characteristics and comparison between groups.

Variable	Total (*n* = 311)	Uncomplicated (*n* = 191)	Complicated (*n* = 120)	*p*-Value
**Age, mean ± SD (years)**	43.28 ± 17.97	39.53 ± 17.58	49.53 ± 16.85	<0.001
**Age range (years)**	18–86	–	–	–
**Male sex, *n* (%)**	182 (58.5%)	112 (58.6%)	70 (58.3%)	0.591
**Female sex, *n* (%)**	129 (41.5%)	79 (41.4%)	50 (41.7%)	–

Abbreviations: SD, standard deviation.

**Table 2:** Multivariable logistic regression analysis of factors associated with complicated appendicitis.

Variable	Adjusted OR	95% CI	*p*-Value
Age (per 10-year increase)	1.25	1.10–1.45	0.002
Male sex	1.10	0.70–1.70	0.65
Leukocyte count (per 1000/mm^3^ increase)	1.05	1.02–1.08	<0.001
C-reactive protein (per 10 mg/L increase)	1.12	1.08–1.16	<0.001

**Abbreviations:** OR, odds ratio; CI, confidence interval; CRP, C-reactive protein.

**Table 3:** Baseline laboratory parameters in patients with uncomplicated and complicated appendicitis (*n* = 311).

Variable	Uncomplicated (*n* = 191)	Complicated (*n* = 120)	*p*-Value
**Leukocyte count, median (IQR) (/mm^3^)**	14,200 (12,100–16,800)	14,800 (12,700–16,500)	0.29
**C-reactive protein (CRP), median (IQR) (mg/L)**	27 (16–48)	31 (19–54)	0.39

Abbreviations: CRP, C-reactive protein; IQR, interquartile range. *Comparisons between groups were performed using the Mann–Whitney U test*.

**Table 4:** Frequency of complicated appendicitis according to age group.

Age Group	*n*	Complicated Appendicitis *n* (%)
**<40 years**	155	48 (31%)
**40–59 years**	92	42 (45.7%)
**≥60 years**	64	30 (46.9%)

Abbreviations: *n* = number of patients. Percentages were calculated within each age group.

## Data Availability

The data presented in this study are available on request from the corresponding author. The data are not publicly available due to patient confidentiality.

## References

[1] Selvaggi L., Pata F., Pellino G., Podda M., Di Saverio S., De Luca G.M., Sperlongano P., Selvaggi F., Nardo B. (2025). Acute appendicitis and its treatment: A historical overview. Int. J. Colorectal Dis..

[2] Sheen J., Bowen J., Whitmore H., Bowling K. (2022). Hyponatremia as a Marker of Complicated Appendicitis: A Retrospective Analysis. Cureus.

[3] Halaseh S.A., Kostalas M., Kopec C., Nimer A. (2022). Bilirubin as a Predictor of Complicated Appendicitis in a District General Hospital: A Retrospective Analysis. Cureus.

[4] Chang Y.J., Chen L.J., Chang Y.J. (2022). Did the severity of appendicitis increase during the COVID-19 pandemic?. PLoS ONE.

[5] Roupakias S., Kambouri K., Al Nimer A., Bekiaridou K., Blevrakis E., Tsalikidis C., Sinopidis X. (2025). Balancing Between Negative Appendectomy and Complicated Appendicitis: A Persisting Reality Under the Rule of the Uncertainty Principle. Cureus.

[6] Michelson K.A., McGarghan F.L.E., Patterson E.E., Waltzman M.L., Samuels-Kalow M.E., Greco K.F. (2022). Clinician factors associated with delayed diagnosis of appendicitis. Diagnosis.

[7] Jumhour H. (2026). Delayed Diagnosis in Outpatient Care: A Systematic Review of Documentation Fragmentation as a Hidden Driver of Diagnostic Error. Cureus.

[8] Bhangu A., Søreide K., Di Saverio S., Assarsson J.H., Drake F.T. (2017). Acute appendicitis: Modern understanding of pathogenesis, diagnosis, and management. Lancet.

[9] Vigil Escalera Bejarano M., Gallardo-Navarro E., Gomez López J.M., Tirado Cortes A.A. (2025). Association Between Patient Age and Severity in Acute Appendicitis. Cureus.

[10] Hui T.T., Major K.M., Avital I., Hiatt J.R., Margulies D.R. (2002). Outcome of elderly patients with appendicitis: Effect of computed tomography and laparoscopy. Arch. Surg..

[11] Shemes A., Elgharib A.A., Elghrieb A., Shetiwy M., Aziz M.A., Elzeftawy S. (2026). Evaluation of the predictive value of scoring systems in diagnosis of acute appendicitis: A comparative prospective study. Langenbecks Arch. Surg..

[12] Mouliou D.S. (2023). C-Reactive Protein: Pathophysiology, Diagnosis, False Test Results and a Novel Diagnostic Algorithm for Clinicians. Diseases.

[13] Bayissa B.B., Miressa F., Abulkadir A., Fekadu G. (2022). Predictors of complicated appendicitis among patients presented to public referral hospitals in Harari region, Eastern Ethiopia: A case-control study. Surg. Pract. Sci..

[14] Liang Y., Sailai M., Ding R., Yimamu B., Kazi T., He M., Liu Z., Lin J., Liu Y., Deng C. (2024). Predictive model for identification of gangrenous or perforated appendicitis in adults: A multicenter retrospective study. BMC Gastroenterol..

[15] Moris D., Paulson E.K., Pappas T.N. (2021). Diagnosis and Management of Acute Appendicitis in Adults: A Review. JAMA.

[16] Podda M., Ceresoli M., De Simone B., Fugazzola P., Pata F., Balla A., Gerardi C., Allocati E., Salminen P., Coimbra R. (2026). Diagnosis and Treatment of Acute Appendicitis: 2025 Edition of the World Society of Emergency Surgery Jerusalem Guidelines. JAMA Surg..

[17] Alajääski J., Lietzén E., Grönroos J.M., Mecklin J.P., Leppäniemi A., Nordström P., Rautio T., Rantanen T., Sand J., Paajanen H. (2022). The association between appendicitis severity and patient age with appendiceal neoplasm histology—A population-based study. Int. J. Colorectal Dis..

[18] Gal M., Maya P., Ofer K., Mansoor K., Benyamine A., Boris K. (2024). Acute Appendicitis in the Elderly: A Nationwide Retrospective Analysis. J. Clin. Med..

[19] Harada T., Harada Y., Hiroshige J., Shimizu T. (2022). Factors associated with delayed diagnosis of appendicitis in adults: A single-center, retrospective, observational study. PLoS ONE.

[20] Lezama M.M., Harriott C.B., Jurado M.G.Á., Aramburu S., Angeramo C.A., Schlottmann F. (2024). Predictors of complicated acute appendicitis: Single-center analysis of a prospective cohort of 2500 adult patients. J. Gastrointest. Surg..

[21] Lapsa S., Ozolins A., Strumfa I., Gardovskis J. (2021). Acute Appendicitis in the Elderly: A Literature Review on an Increasingly Frequent Surgical Problem. Geriatrics.

[22] Schrempf M.C., Schiele S., Anthuber M., Anthuber L., Hoffmann M., Sommer F., Mair A. (2026). Safety of In-Hospital Delay of Appendectomy in Elderly Patients-A Retrospective Analysis of 525 Consecutive Patients Aged 65 and Older Undergoing Surgery for Suspected Appendicitis. World J. Surg..

[23] Ribeiro A.M., Romero I., Pereira C.C., Soares F., Gonçalves Á., Costa S., da Silva J.B. (2022). Inflammatory parameters as predictive factors for complicated appendicitis: A retrospective cohort study. Ann. Med. Surg..

[24] Naderan M., Babaki A.E., Shoar S., Mahmoodzadeh H., Nasiri S., Khorgami Z. (2016). Risk factors for the development of complicated appendicitis in adults. Ulus. Cerrahi Derg..

[25] Drake F.T., Mottey N.E., Farrokhi E.T., Florence M.G., Johnson M.G., Mock C., Steele S.R., Thirlby R.C., Flum D.R. (2014). Time to appendectomy and risk of perforation in acute appendicitis. JAMA Surg..

[26] van Dijk S.T., van Dijk A.H., Dijkgraaf M.G., Boermeester M.A. (2018). Meta-analysis of in-hospital delay before surgery as a risk factor for complications in patients with acute appendicitis. Br. J. Surg..

[27] Renteria O., Shahid Z., Huerta S. (2018). Outcomes of appendectomy in elderly veteran patients. Surgery.

[28] Bălănescu L., Băetu A.E., Cardoneanu A.M., Moga A.A., Bălănescu R.N. (2022). Predictors of Complicated Appendicitis with Evolution to Appendicular Peritonitis in Pediatric Patients. Medicina.

[29] Ghali M.S., Hasan S., Al-Yahri O., Mansor S., Al-Tarakji M., Obaid M., Shah A.A., Shehata M.S., Singh R., Al-Zoubi R.M. (2023). Adult appendicitis score versus Alvarado score: A comparative study in the diagnosis of acute appendicitis. Surg. Open Sci..

[30] Şimşek O., Şirolu S., Özkan Irmak Y., Hamid R., Ergun S., Kepil N., Tutar O. (2024). Comparative study of imaging features in uncomplicated and complicated acute appendicitis. Ulus. Travma Acil Cerrahi Derg..

[31] Iamwat J., Teerasamit W., Apisarnthanarak P., Noppakunsomboon N., Kaewlai R. (2021). Predictive ability of CT findings in the differentiation of complicated and uncomplicated appendicitis: A retrospective investigation of 201 patients undergone appendectomy at initial admission. Insights Imaging.

[32] Barbu L.A., Cercelaru L., Vîlcea I.-D., Șurlin V., Mogoantă S.-S., Țenea Cojan T.S., Mărgăritescu N.-D., Popescu M., Mogoș G.F.R., Vasile L. (2025). Coexistence of Acute Appendicitis and Mesenteric Cystic Lymphatic Malformation in an Adult: A Case Report and Narrative Review of Intraoperative Management Strategies. Life.

[33] Vasile L., Barbu L.A., Mogoş G.F.R., Şurlin V., Vîlcea I.D., Cercelaru L., Mogoantă S.Ş., Mărgăritescu N.D., Nimigean V., Nimigean V.R. (2025). Neuroendocrine tumors of the appendix: A comprehensive review of the literature and case presentation. Rom. J. Morphol. Embryol..

[34] Souza I.M.A.G., Nunes D.A.A., Massuqueto C.M.G., Veiga M.A.M., Tamada H. (2017). Complicated acute appendicitis presenting as an abscess in the abdominal wall in an elderly patient: A case report. Int. J. Surg. Case Rep..

